# Lumbosacral spinal cord functional connectivity at rest: From feasibility to reliability

**DOI:** 10.1162/imag_a_00286

**Published:** 2024-09-05

**Authors:** Ilaria Ricchi, Nawal Kinany, Dimitri Van De Ville

**Affiliations:** Neuro-X Institute, Ecole Polytechnique Fédérale de Lausanne (EPFL), Geneva, Switzerland; Department of Radiology and Medical Informatics, University of Geneva, Geneva, Switzerland

**Keywords:** lumbosacral spinal cord fMRI, functional connectivity, PNM-based pipelines, robustness and reliability

## Abstract

In the past decade, exploration of spontaneous blood-oxygen-level-dependent (BOLD) signal fluctuations has expanded beyond the brain to include the spinal cord. While most studies have predominantly focused on the cervical region, the lumbosacral segments play a crucial role in motor control and sensory processing of the lower limbs. Addressing this gap, the aims of the current study were twofold: first, confirming the presence and nature of organized spontaneous BOLD signals in the human lumbosacral spinal cord; second, systematically assessing the impact of various denoising strategies on signal quality and functional connectivity (FC) patterns. Given the susceptibility of spinal cord functional magnetic resonance imaging (fMRI) to noise, this step is pivotal to ensure the robustness of intrinsic FC. Our findings uncovered bilateral FC between the ventral and dorsal horns. Importantly, these patterns were consistently observed across denoising methods and demonstrating fair to excellent split-half temporal stability. Importantly, the evaluation of diverse denoising strategies highlighted the efficacy of physiological noise modeling (PNM)-based pipelines in cleaning the signal while preserving the strength of connectivity estimates. Together, our results provide evidence of robust FC patterns in the lumbosacral spinal cord, thereby paving the way for future studies probing caudal spinal activity.

## 1 Introduction

Functional magnetic resonance imaging (fMRI) is a noninvasive imaging technique that has revolutionized our ability to investigate the central nervous system (CNS). By exploiting the blood-oxygenation-level-dependent (BOLD) signal, a hemodynamic proxy of neural activity (Logothetis, 2003), fMRI has become a method of choice for exploring brain function. Notably, the acquisition of fMRI data during resting state—marked by the absence of explicit tasks or stimuli—has garnered significant attention. This interest stems from the compelling observation that spontaneous BOLD fluctuations can be parsed into so-called resting-state networks (Biswal et al., 2010;Damoiseaux et al., 2006;Fox & Raichle, 2007), which reflect the brain’s intrinsic functional organization. These networks have been shown to be behaviorally relevant, making them valuable tools to study healthy and impaired brain function (van den Heuvel & Hulshoff Pol, 2010).

More recently, the scope of resting-state fMRI has expanded beyond the confines of the cortex, for instance to probe the intrinsic organization of the spinal cord; see (Harrison et al., 2021) for review. Notably, studies focusing on the cervical spinal cord have uncovered organized spontaneous signals, utilizing both data-driven methods, such as independent component analysis (ICA) (Kong et al., 2014;Landelle et al., 2021), and innovation-driven coactivation pattern analysis (iCAP) (Kinany et al., 2020), and hypothesis-driven approaches (Barry et al., 2014;Eippert, Kong, Winkler, et al., 2017;Harita & Stroman, 2017;Kaptan et al., 2023;Liu et al., 2016;Weber et al., 2018). These studies have effectively revealed spinal resting-state networks, prominently featuring functional connectivity between bilateral ventral (i.e., motor) and dorsal (i.e., sensory) horns. Building on these auspicious results, further studies have then explored the reliability of these functional connectivity patterns, a critical consideration to ensure their broader applicability in fundamental and clinical applications. For instance, Barry and colleagues demonstrated their stability within the same scanning session (Barry et al., 2016). Additional investigations have evaluated the impact of different acquisition and processing choices (Barry et al., 2018;Eippert, Kong, Winkler, et al., 2017;Kinany et al., 2023), as well as the influence of distinct noise sources (Kaptan et al., 2023). These collective efforts have underscored the robustness of functional connectivity patterns in the cervical spinal cord.

Despite these promising findings, areas situated caudal to the cervical spinal cord have so far largely eluded investigation. Remarkably, the lumbosacral spinal cord, crucial for motor control and sensory processing of the lower limbs, has been largely unexplored. This lack of studies can be attributed, in part, to the challenges associated with fMRI acquisition, processing, and analysis in this region, stemming from the smaller size of the cord (Frostell et al., 2016), heightened anatomical variability (Tins & Balain, 2016;Van Schoor et al., 2015), and the lack of dedicated tools have compounded this difficulty. Nonetheless, it is interesting to note that the lumbosacral cord has a greater proportion of gray matter compared with cervical segments (Henmar et al., 2020). To date, only one study (Combes et al., 2023) has deployed resting-state fMRI to uncover the intrinsic organization of the lumbosacral spinal cord, shedding light on sensorimotor networks reminiscent of those observed in the cervical spinal cord. Drawing on these promising observations, a critical aspect yet to be explored pertains to the robustness of these lumbosacral functional connectivity patterns. Addressing this is pivotal for the advancement of resting-state metrics as potent tools to identify functional biomarkers, especially in the context of neurological conditions such as movement disorders and spinal cord injuries (Conrad et al., 2018;Kreiter et al., 2022;Rowald et al., 2022).

To address this knowledge gap, this study sets out to systematically investigate spontaneous BOLD fluctuations in the human lumbosacral spinal cord. Recognizing the susceptibility of spinal cord fMRI to a variety of noise sources, our aims are twofold: firstly, to confirm the presence and nature of organized fluctuations in the lumbosacral spinal cord, employing a sequence distinct from the one used byCombes et al. (2023); and secondly, to assess the robustness of functional connectivity measures to variations in the denoising procedure. By identifying the optimal approach for assessing functional connectivity in the lumbosacral spinal cord, we intend to contribute to the development of robust methods for studying the functional architecture of this region. Ultimately, our work will enhance our ability to investigate the CNS on a larger scale, opening up new avenues for research and clinical applications.

## 2 Methods

### 2.1 Participants

In total, 22 healthy volunteers were enrolled in this study (14 male, 14 female, 28 ± 2.28 years old). Participants reported no history of neurological or motor disorders. All participants gave their written informed consent to participate, and the study was approved by the Commission Cantonale d’Éthique de la Recherche Genève (CCER, study 2019-00203).

### 2.2 Data acquisition

All experiments were performed on a Siemens Prisma scanner (3 Tesla) (Erlangen, Germany), equipped with a 32-channel spine coil of which 16 were used for the acquisitions. Participants were placed in the scanner in supine position. Functional images were acquired using a T2*-weighted echo-planar imaging (EPI) sequence with ZOOMit selective field-of-view imaging (see example image inFig. 1A), based on our previous cervical protocols (Kinany et al., 2019,^2020^,^2023^), but adapted for the lumbosacral spinal cord (repetition time (TR) = 2.5 s, echo time (TE) = 34 ms, FOV = 44 × 144, flip angle = 80°, GRAPPA acceleration factor: 2, in-plane resolution = 1.1 × 1.1 mm^2^, slice thickness = 3 mm). Compared with cervical recordings, a wider field-of-view (i.e., changing in-plane resolution from 1 to 1.1 mm) was employed to account for the additional tissue volume present at the levels of the hips, and to avoid aliasing artifacts. The lumbosacral enlargement (approximately from at vertebral levels T11 to L2) was covered using 27 axial slices, positioned perpendicularly to the spinal cord to limit signal dropouts due to field inhomogeneities (Finsterbusch et al., 2012). Manual shimming adjustments focused on the spinal cord were conducted prior to the functional acquisitions to optimize the magnetic field homogeneity. For each participant, 360 volumes (i.e., 15 min) were acquired, during rest (i.e., no explicit task) with eyes open (an empty screen was shown). Additionally, high resolution T2-weighted images (64 sagittal slices; resolution: 0.4 × 0.4 × 0.8 mm^3^; field-of-view: 250 × 250 mm^2^; TE: 133 ms; flip angle: 140°; TR: 1500 ms; GRAPPA acceleration factor: 2; partial Fourier factor: 6/8; acquisition time: 6:03 min) were acquired for normalization purposes.

**Fig 1.**
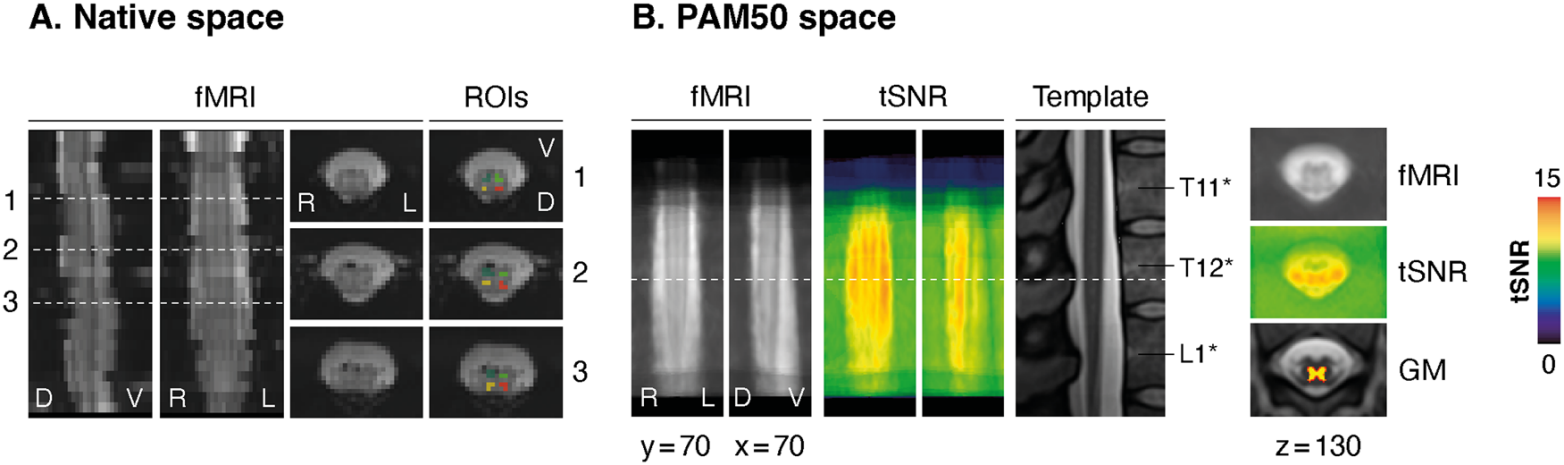
Data quality. (A) Mean functional image (fMRI) for an example participant in native space. Left: Sagittal and coronal views. Right: Three axial views (slice position indicated by dashed lines). The last column shows ROI masks overlaid on axial views of the mean functional image. (B) Left: Coronal and sagittal views of the mean normalized functional image across all participants. Panels for tSNR display average values (conventional PNM + Moco + CSF denoising). A sagittal view of the T2w-PAM50 template is included for reference, with labeled vertebral bodies. Right: Corresponding axial views (slice position indicated by dashed line) with gray matter (GM) mask overlaid on the template image.

During the fMRI data acquisition, we recorded peripheral physiological signals to perform physiological noise modeling: cardiac data were acquired using a photoplethysmograph and respiratory signals were obtained with a belt (Biopac MP150 system, California, USA). Simultaneous recordings of scanner triggers ensured synchronization of the recordings.

### 2.3 Data preprocessing

Preprocessing steps were performed using Python (version 3.9,7), with nilearn library (version 0.9.1) falling under the umbrella of scikit-learn (version 0.24.2), FMRIB Software Library (FSL; version 5.0) and Spinal Cord Toolbox (SCT; version 5.3.0;De Leener et al., 2017).

#### 2.3.1 Preprocessing of fMRI data

The first step of the preprocessing pipeline was slice-timing correction of the functional volumes, using the FSL command “slicetimer.”

##### 2.3.1.1 Motion correction

Given the small size of the spinal cord, in particular at the lumbosacral levels (Frostell et al., 2016), motion correction is a crucial step. The volumes of each functional run were averaged and the centerline of the spinal cord was automatically extracted from the resulting image. A cylindrical mask along this centerline was drawn (30 mm) and further used to exclude regions outside the spinal cord, thus limiting the impact of regions that might move independently of the cord. To account for the articulated structure of the spine, in-plane slice-wise realignment (in x and y) was then performed using the SCT function*“sct_fmri_moco”*(De Leener et al., 2017), with no z-regularization and a B-spline interpolation. Motion correction parameters were computed as the average absolute motion across slices and subsequently employed as regressors for the denoising strategy (seeSection 2.3.2.). To provide an overall estimate of motion between each time point, framewise displacement (FD) was computed by summing the absolute values of the derivatives of the motion parameters in x and y. The mean FD was then used to determine which subjects to exclude from the analyses.

##### 2.3.1.2 Segmentation

For the anatomical image, the spinal cord was automatically segmented with SCT deep learning model (sct_deepseg -task lumbar) and the masks were manually adjusted.

For the functional runs, we use the FSLeyes software to manually create binary masks of the spinal cord only and the spinal cord with the surrounding subarachnoid cavity, using mean motion-corrected images. The subtraction of the former from the latter generated the mask of the CSF only, which was manually inspected for each participant.

##### 2.3.1.3 Normalization

Functional images were first coregistered to the corresponding T2 anatomical image with nonrigid transformations, using the SCT function “sct_register_multimodal” (De Leener et al., 2017). Normalization warping fields from anatomical image to PAM50 template space were also estimated. Vertebrae-based alignment, standardly used for cervical images, is suboptimal in the lumbosacral region due to its smaller size and to large shifts between spinal segmental levels and vertebral bodies (Frostell et al., 2016). To circumvent this issue, we placed a label on the conus medullaris (tip of the cord, at the beginning of the cauda equina), which was used as reference for alignment instead. The SCT function “sct_register_to_template” was used to straighten the spinal cord along its centerline and normalize it to the PAM50 template (De Leener et al., 2017) using nonrigid registration. The warping fields obtained for the coregistration (functional images in the anatomical space) and normalization (anatomical images in the template space) were concatenated to generate warping fields from the functional to the template space. The warping field template-to-functional was then applied (using the function “sct_apply_transfo”) to the PAM50 gray matter probability maps (thresholded across subjects at a value that preserved 50% of the probability distributions and binarized), to bring them into the native space where subsequent analyses were carried out. This was used to define regions-of-interest (ROIs) corresponding to each horn, ensuring a gap of one voxel between them (see examples inFig. 1A).

#### 2.3.2 Denoising and temporal filtering

We systematically investigated the impact of distinct denoising procedures in the lumbosacral spinal cord by applying denoising pipelines incorporating different confounds. A temporal band-pass filter (cutoff frequencies: 0.01 Hz and 0.13 Hz) was applied. Each denoising procedure relies on the usage of the*“clean_img”*function from nilearn library, which allows us to remove the noise confounds orthogonally to the temporal filter. Specifically, confounds and temporal filter were projected onto the same orthogonal space, following the methodology outlined inLindquist et al. (2019), instead of being applied sequentially.

Physiological data were used to build nuisance regressors, using a model-based approach derived from the RETROspective Image CORrection (RETROICOR) procedure (Glover et al., 2000). This model assumes the physiological signals to be quasi-periodic, which leads to uniquely assigning the cardiac and respiratory phases to each image using a Fourier expansion. To this aim, we resorted to FSL’s physiological noise modeling (PNM) tool to generate regressors from cardiac, respiratory, and CSF signals. Cardiac peaks were automatically detected using the*“scipy.signal.find_peaks”*function (Virtanen et al., 2020), with manual inspection to ensure reliability.

We followed recommendations for PNM in the spinal cord (Kong et al., 2012). For both cardiac and respiratory regressors, we employed an order of 4, which means that the base frequency was used along with the first three harmonics. Cardiac and respiratory signals were combined with an interaction order of 2. This resulted in a total of 32 slice-wise regressors. A CSF regressor was also calculated as the mean signal from the top 10% of CSF voxels with the most signal variability. Of note, for these slice-wise regressors, the*“clean_img”*function was applied independently on each slice.

In addition to model-based denoising approaches, we leveraged the CSF signal to account for non-neural fluctuations using a data-driven method. Specifically, we used a component-based noise correction technique known as CompCor (Behzadi et al., 2007), which estimates K regressors (K set to 5 in our case) corresponding to the most significant principal components derived from CSF noise. We implemented this method using the*“nipype.algorithms.confounds”*module.

The combination of all the above mentioned procedures resulted in 12 denoising pipelines illustrated inTable 1.

**Table 1.** Denoising pipelines.

	Baseline	CSF	Milton parameters	CompCor	Cardiac	Respiratory	Card-resp interaction	# regressors
Baseline								0
CSF		✓						1
Moco			✓					2
CompCor				✓				5
Moco + CompCor			✓	✓				7
Cardiac					✓			8
Respiratory						✓		8
PNM					✓	✓	✓	32
PNM + CSF		✓			✓	✓	✓	33
PNM + Moco			✓		✓	✓	✓	34
PNM + Moco + CSF		✓	✓		✓	✓	✓	35
All		✓	✓	✓	✓	✓	✓	40
*# regressors (per option)*	0	1	2	5	8	8	16	

This table depicts the nuisance regressors taken into account (columns) for each denoising pipeline (rows), along with the count of regressors of interest per option (last row) and the overall total considering the several combinations (last column).

#### 2.3.3 Temporal SNR (tSNR) and explained variance

To evaluate the impact of the different denoising procedures on the signals, we calculated for each participant the temporal signal-to-noise ratio (tSNR) and the explained variance of the time series within a mask combining horns across slices (seeSection 2.4). The voxelwise tSNR values were obtained with the SCT’s function “*sct_fmri_compute_tsnr”*(De Leener et al., 2017) which computes each voxel’s temporal mean and divides it by its standard deviation. TSNR values were also averaged in the four gray matter ROIs. The explained variance (R^2^) was computed as the fractional reduction of signal variance (Birn et al., 2014):

$$R^{2}\;\;=1-\frac{\sigma_{denoised}}{\sigma_{baseline}}$$,

where$\sigma_{denoised}$is the variance of the denoised signals for a specific denoising procedure and$\sigma_{baseline}$indicates the baseline variance of the time series before denoising (i.e., after motion correction). The R^2^values were then adjusted to take into account the number of regressors used in the denoising procedure:

$$Adjusted\;R^{2}\;=\left\{1-\left[\frac{\left(1-R^{2})\;(n-1\right)}{\left(n-k-1\right)}\right]\right\}$$,

wherenrepresents the number of time points andkthe number of regressors in the nuisance model.

As the Kolmogorov–Smirnov test rejected the normality assumption for the tSNR and R^2^distributions, we resorted to the Welch’s*t*-test, which is robust for small sample size and non-normality (Ahad & Syed-Yahaya, 2014). In particular, we compared the explained variance distributions in order of increasing complexity: comparing each denoising method against the previous, less complex, strategy (namely, Moco with CSF, CompCor with Moco, etc.). As for the tSNR, we also relied on Welch’s*t*-test, this time to compare each denoising procedure distribution with respect to the baseline.

### 2.4 Data analyses

**Fig. 2.**
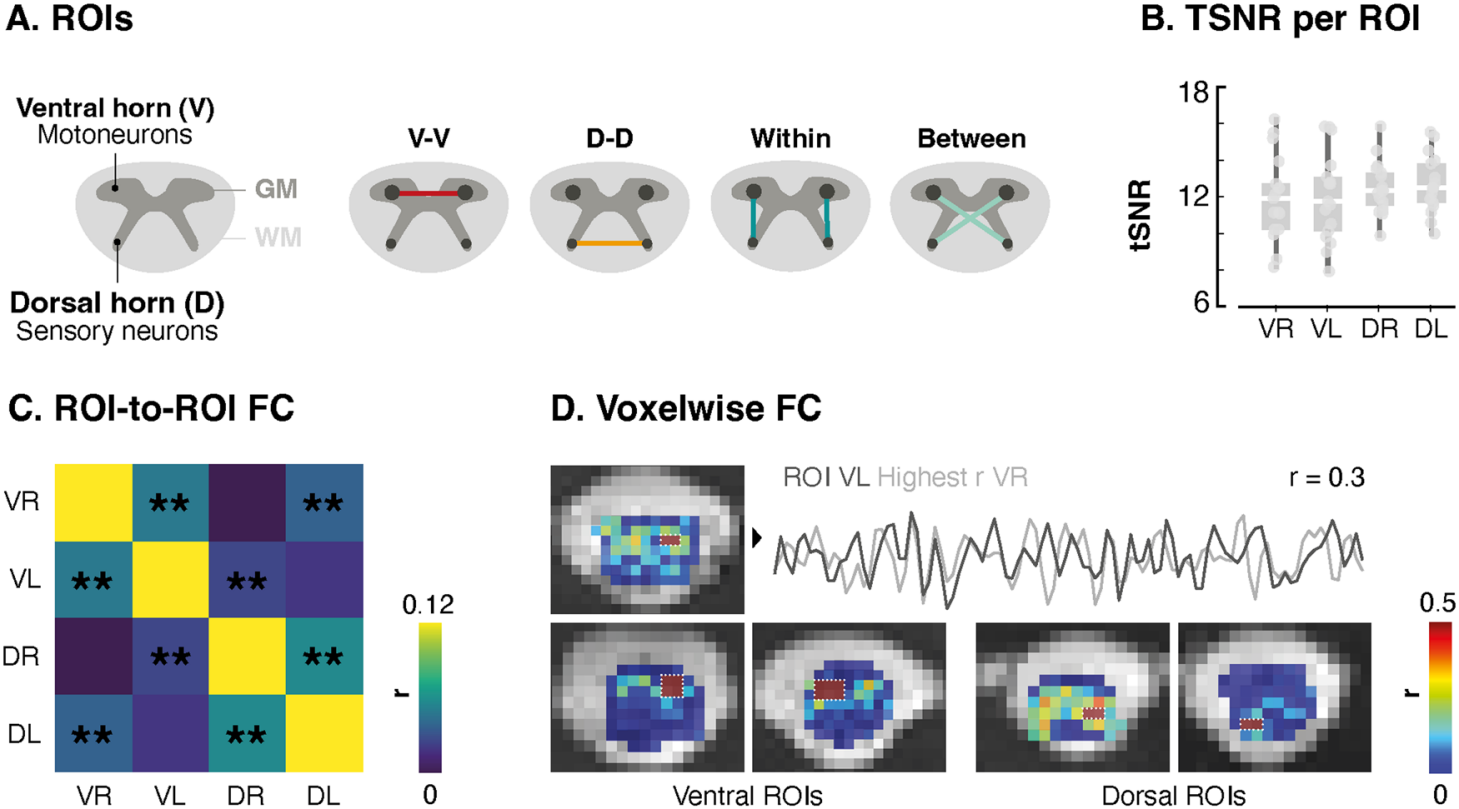
Extension of resting-state FC to the lumbosacral cord. (A) Schematic cross sections of the spinal cord. The left panel illustrates the spinal cord’s structure, featuring the characteristic butterfly-shaped gray matter (GM) surrounded by white matter (WM). The GM can be divided into four horns, housing motor (ventral horns, V) and sensory (dorsal horns, D) neurons. The right panel outlines potential connectivity patterns between these four regions of interest (ROIs). (B) TSNR in the four ROIs, for the*PNM + Moco + CSF*denoising strategy. Each box represents the distribution (i.e., from the 25th to the 75th percentile) of tSNR values across participants, with medians represented by the horizontal white line inside the box. Vertical lines correspond to the 1.5 interquartile range and dots represent tSNR values for each of the 17 participants. (C) 4 x 4 correlation matrix showing FC between the four horns (ROI-to-ROI analysis, for the*PNM + Moco + CSF*denoising strategy). Significant connectivity is observed between bilateral ventral horns (r = 0.05), between bilateral dorsal horns (r = 0.06), and between bilateral dorsoventral horns (r = 0.032). (D) Example slices showcasing results from the ROI-to-voxels analysis, for the*PNM + Moco + CSF*denoising strategy. The voxels of the ROIs are outlined with a dotted line. Resulting correlations (i.e., other voxels) overlaid on the mean functional image. In the top row, correlation patterns for an ROI in the left ventral (VL) horn are shown. The highest correlation is observed in the contralateral ventral horn (r = 0.3). Corresponding time courses are presented. The bottom row presents additional connectivity maps for ventral ROI on the left panel, while examples for dorsal ROI are presented on the right panels. VR = ventral right, VL = ventral left, DR = dorsal right, DL = dorsal left.

Functional connectivity (FC) analyses were performed in the native functional space of the participants, using a ROI-based approach. For each slice, ROIs in four specific locations were used (ventral and dorsal horns on both sides,Fig. 2A).

Static functional connectivity was estimated by means of Pearson correlation coefficients and two types of analyses were conducted: (i) ROI-to-ROI and (ii) ROI-to-voxels.

#### 2.4.1 ROI-to-ROI functional connectivity

Functional connectivity was computed using a region of interest (ROI)-based approach where we considered the four ROIs to extract ROI-specific time courses (i.e., average time course for each ROI, slice, and participant). This methodology aligns with earlier work in the cervical spinal cord (Barry et al., 2014;Eippert, Kong, Jenkinson, et al., 2017;Kaptan et al., 2023;Kinany et al., 2019;Kong et al., 2014;Weber et al., 2016). We computed slice-wise Pearson correlation coefficients between those time courses, resulting in a 4 x 4 matrix that summarizes the connectivity patterns of interest: ventral-ventral (VV), dorsal-dorsal (DD), within (W), and between (B) hemicords (Fig. 2A). Slice-wise correlation coefficients were then averaged over slices to yield one 4 x 4 matrix per participant. We performed this analysis across all the denoising procedures to compare the impact of each strategy on functional connectivity estimates.

The significance of functional connectivity estimates was assessed using nonparametric tests. Indeed, even after applying a Fisher z-transformation to the correlation values, the Kolmogorov–Smirnov test rejected the assumption of normal distributions. Consequently, the Wilcoxon test was conducted on the correlation values to evaluate which connectivity pattern was significantly different from zero using the different denoising techniques (corrected for multiple comparison with Benjamini–Hochberg method).

#### 2.4.2 ROI-to-voxels functional connectivity

**To further explore ROI-based correlations**, functional connectivity was estimated within each slice, focusing on a single ROI at a time. The correlations between the average time courses of this ROI and those of each voxel within the slice were computed. This enabled visual assessment of FC patterns, similar to earlier work (Barry et al., 2014). For the sake of brevity, we present these results exclusively for time series processed using the denoising approach typically used in spinal cord fMRI (i.e.,*PNM + Moco + CSF*).

#### 2.4.3 Split-half temporal stability

To investigate the temporal stability of lumbosacral resting-state functional connectivity patterns, we split the fMRI time series of each participant into two halves in which correlation values were independently extracted. We computed the intraclass correlation coefficient (ICC) to measure the stability of functional connectivity estimates across the temporal splits, for each denoising procedure. For this analysis, we used a two-way random effect model, “Case 2” intraclass correlation coefficient defined as

$$ICC(2,1)=\frac{\sigma_{\;\;\;\;between}^{2}}{\sigma_{\;\;\;\;between}^{2}+\sigma_{\;\;\;\;session}^{2}+\sigma_{\;\;\;\;error}^{2}},$$

where$\sigma_{\;\;\;\;between}^{2}$corresponds to the variance between participants and$\sigma_{\;\;\;\;session}^{2}$indicates the variance between sessions (i.e., the two halves). This metric, known as “absolute agreement” in the literature, quantifies the proportion of total variance attributed to between-participants differences (Kaptan et al., 2023;McGraw & Wong, 1996;Shrout & Fleiss, 1979). To assess the uncertainty of this metric, we calculated the 95% confidence interval (CI) of the ICC values using a bootstrap procedure implemented in Python with the*pingouin*library (Vallat, 2018). According to established standards (Cicchetti & Sparrow, 1981;Hallgren, 2012), the ICC values are interpreted as follows: poor <0.4, fair 0.4–0.59, good 0.6–0.74, excellent ≥0.75.

To have a direct comparison between split-half and full connectivity (only for the standard PNM + Moco + CSF pipeline), we also tested whether FC distribution for each connectivity pattern in the split-half datasets differed significantly from those derived from the full timeseries (Kruskal–Wallis test).

In addition to investigating the temporal stability of functional connectivity estimates, we also calculated ICC in the split-half datasets for the following metrics: (i) CSF, (ii) cardiac, and (iii) respiratory. For (i) CSF, we computed the mean amplitude of the signal from the power spectral density of each half of the regressor. For (ii) cardiac, we computed the average difference between cardiac peaks. For (iii) respiratory, respiration traces were first band-pass filtered (cutoff frequencies: 0.01 Hz and 0.6 Hz), and median filtered over 1 s. Subsequently, we identified the respiratory cycle by applying a Hilbert transform and by computing the phase of the signals (which measures the position of a waveform in time). We then determined the occurrence of zero-crossings within the detected respiratory cycles.

## 3 Results

### 3.1 Functional data quality control

Out of the initial 28 participants, 4 were excluded from the study due to excessive motion (i.e., average FD >0.4 mm). InFigure 1B, we present the mean functional image for the remaining 24 participants, normalized to the PAM50 space, along with the corresponding tSNR map (using the conventional PNM + Moco + CSF denoising scheme). The distinctive butterfly shape of the gray matter is clearly discernible, confirming the accuracy of the normalization procedure. The tSNR values are presented independently for each of the four horns (Fig. 2B): ventral right =11.93 (8.23–15.41) (median across participants and interquartile range, IQR), ventral left =11.80 (8.09–15.74), dorsal right =11.88 (10.04–14.36), dorsal left =12.03 (10.06–15.15).

### 3.2 Extending resting-state FC to the lumbosacral spinal cord

A main goal of this study was to extend resting-state FC fMRI findings beyond the cervical spinal cord, by deploying such analyses at the lumbosacral level. To this end, we investigated connectivity patterns between the ventral and dorsal horns of both left and right hemicords (Fig. 1C). Using this ROI-based approach, we observed a significant positive correlation between bilateral ventral horns (median correlation value r = 0.05, Wilcoxon test, W = 3, p < 0.004, Benjamini–Hochberg corrected) and between dorsal horns (r = 0.06, W = 5, p < 0.004). Additionally, dorsal-ventral connectivity between hemicords was also significantly positive (r = 0.032, W = 87, p < 0.004). Within-hemicord connectivity, instead, was nonsignificant (r = 0.017, p = 0.08). When assessing the robustness of these connectivity estimates in each participant, we observed that ventral and dorsal connectivities were positive in 70.8% and 83.3% of participants, respectively. Overall, 62.5% exhibited positive dorsal-ventral within-hemicord connectivity, and 72.9% exhibited positive dorsal-ventral between-hemicord connectivity.

Evidence of ventral and dorsal connectivity patterns, both within and between hemicords, was also observed using an ROI-to-voxels analysis (Fig. 2D).

### 3.3 Impact of denoising strategies on signal properties

Given the inherent sensitivity of spinal cord fMRI to various noise sources (e.g., breathing, heart rate, motion,…), we systematically and quantitatively compared the impact of different denoising techniques.

**Fig. 3.**
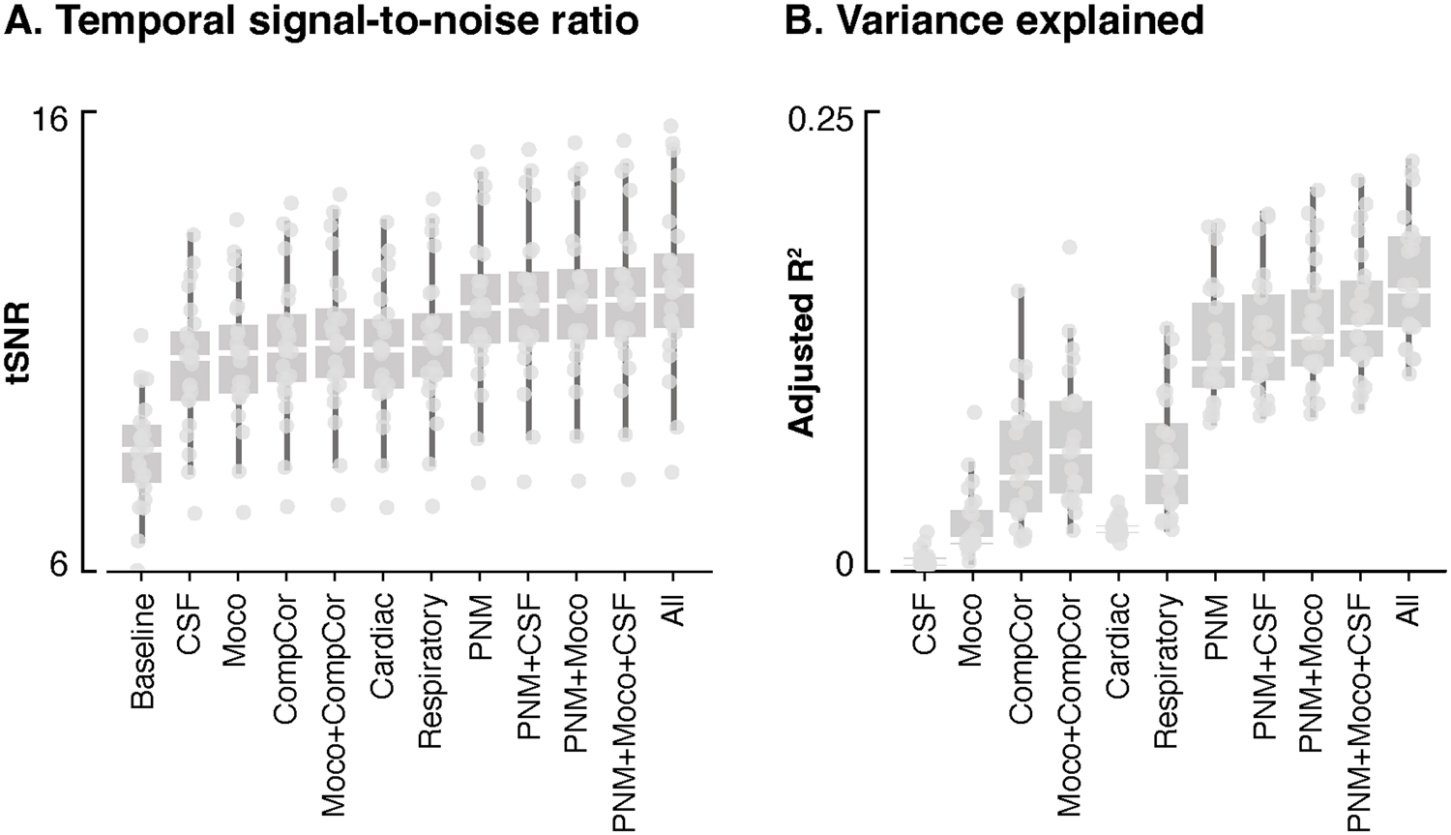
Signal properties following distinct denoising strategies. Temporal signal-to-noise ratio (tSNR) (A) and variance explained (adjusted R^2^) (B) values (y-axis) are presented for each denoising strategy (x-axis). The boxes represent the interquartile range (IQR), spanning from the 25th to the 75th percentile of the data, with the horizontal white line within each box indicating the median value across participants. Each dot represents the average metric (tSNR and adjusted R^2^) of the voxels within the mask combining horns across slices for a specific participant.

First, we assessed changes in signal quality, by evaluating tSNR and variance explained (i.e., adjusted R^2^) for each applied strategy (Fig. 3). We observed that the addition of nuisance regressors led to an increase of tSNR, with all denoising approaches significantly increasing the tSNR compared with the*baseline*pipeline (p < 0.001, Welch’s*t*-test corrected for multiple comparisons). The largest changes were observed when going from the*baseline*pipeline to mild denoising (e.g.,*CSF*or*Moco*pipelines, 24% and 24.5% increase compared with the baseline, respectively), and when adding the PNM-related regressors (e.g., 31% for the*PNM*pipeline). The tSNR reaches its highest value for the most stringent denoising technique (i.e., with all regressors combined) (12.13 (9.3–15.3), median across participants (IQR), 34% increase compared with*baseline*). As for R^2^, the peak was also observed when combining all regressors. Generally, methods incorporating PNM regressors (*PNM*: 0.11 (0.08–0.19),*PNM + CSF:*0.12 (0.09–0.19),*PNM + Moco:*0.13 (0.9–0.19),*PNM + CSF + Moco:*0.13 (0.1–0.2),*PNM + CSF + Moco + CompCor:*0.15 (0.12–0.22), median R^2^across participants (IQR)) explained more variance than those that did not (*CSF:*0.005 (0.001–0.015),*Moco:*0.02 (0.01–0.06),*CompCor:*0.05 (0.02–0.11),*Moco + CompCor:*0.03 (0.03–0.13),*Cardiac:*0.022 (0.02–0.032),*Respiratory:*0.05 (0.03–0.12)). When comparing procedures sequentially, we observed that*Cardiac*exhibited an R^2^significantly lower than the surrounding approaches (*Moco + PNM*and*Respiratory*) and there is a significant improvement when using PNM with respect to respiratory (p < 0.001, Welch’s*t*-test corrected for multiple comparisons).

### 3.4 Impact of denoising strategies on functional connectivity

**Fig. 4.**
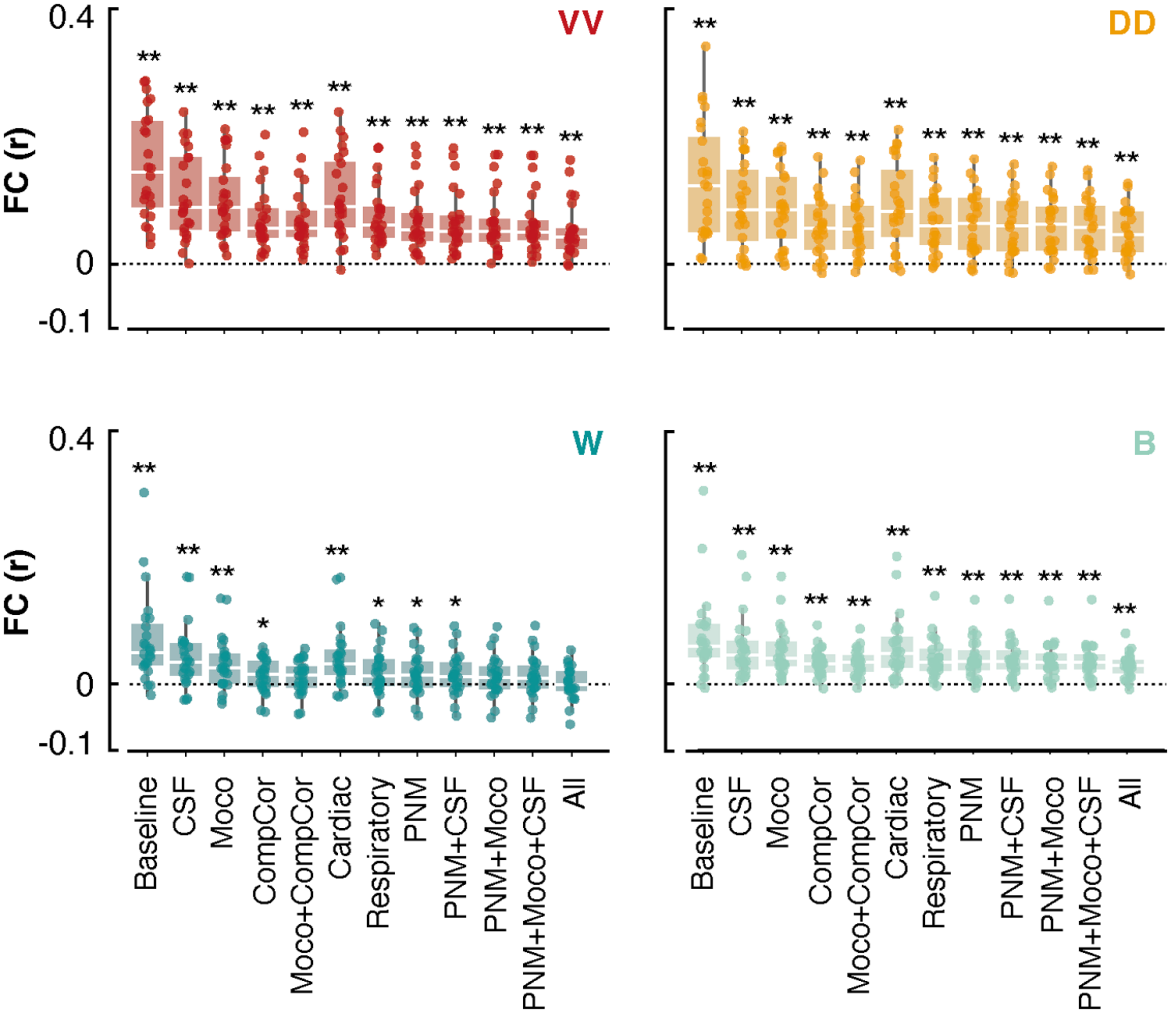
Static functional connectivity using distinct denoising strategies. For each ROI-to-ROI pattern (Fig 2A), we present functional connectivity estimates (y-axis) for each denoising strategy (x-axis). The boxes represent the interquartile range (IQR), spanning from the 25th to the 75th percentile, with the horizontal white line within each box indicating the median value across participants. Each dot represents the mean FC (across slices) for a specific participant. *Indicates p < 0.05 and **p < 0.01.

We then focused on assessing the different denoising procedures from the perspective of functional connectivity (Fig. 4). Connectivity between bilateral ventral (VV) and dorsal (DD) horns, as well as between hemicords (B), appeared to be significant, regardless of the denoising method employed. In contrast, FC within hemicords (W) was not significant for four pipelines (Moco + CompCor, PNM + CSF, PNM + Moco + CSF, and All). For all conditions, the highest connectivity values were obtained for the nondenoised time series (VV: 0.14 (0.04–0.28), DD: 0.12 (0.01–0.26), W: 0.05 (-0.001–0.19), B: 0.06 (0.002–0.2), median across participants (IQR)), as well as for the images denoised with the*Cardiac*pipeline (VV: 0.09 (0.02–0.21), DD: 0.08 (-0.005–0.19), W: 0.03 (-0.017–0.15), B: 0.05 (0.005–0.16). Stricter denoising procedures led to reduced functional connectivity, with connectivity being the weakest for the*All*pipeline (VV: 0.04 (0.001–0.14), DD: 0.05 (-0.004–0.12), W: -0.001 (-0.04–0.04), B: 0.03 (0.001–0.06)).

### 3.5 Temporal stability

**Fig. 5.**
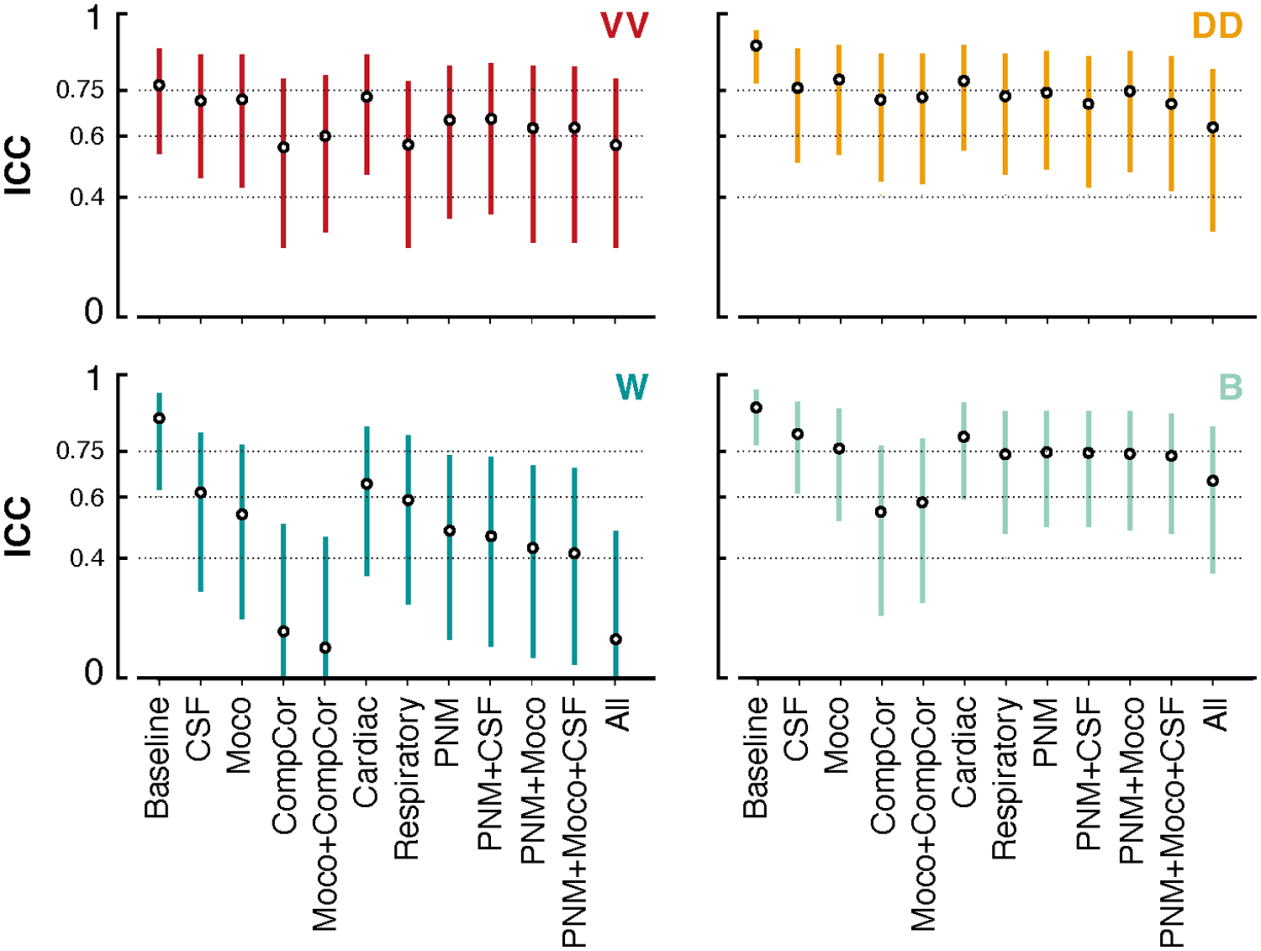
Split-half temporal stability using ICC. Distribution of ICC (Interclass Correlation) scores, depicting the temporal stability of functional connectivity patterns derived from the fMRI signals of each participant. Each point in the figure represents the ICC score for a specific denoising technique employed in the analysis (x-axis), and the bars represent the confidence intervals at 95%. The dotted lines indicate the ICC ranges: poor <0.4, fair 0.4–0.59, good 0.6–0.74, excellent ≥0.74.

Finally, we investigated the split-half temporal stability of the connectivity estimates (Fig. 5, seeFig. S1for scatter plots andFig. S2for a comparison between split-half FC values and those derived from the full dataset). In general, VV, DD, and B connectivity demonstrated a high stability, predominantly within the good range (VV = 0.65, DD = 0.74, B = 0.73, on average over denoising strategies). ICC estimates for W showed a broader distribution (ICC = 0.45 on average), with one value in the excellent range, two in the good range, and six and three in the fair and poor ranges, respectively. For all connectivity patterns, a similar ICC profile was observed, albeit with different amplitudes. Notably, the baseline denoising strategy consistently exhibited the highest temporal stability, achieving an excellent rating. For VV, W, and B connectivity patterns, strong declines in ICC were observed when removing*CompCor*regressors, which brought reliability in the fair (VV and B) and poor (W) ranges. For W, applying the All pipeline also resulted in an ICC value in the poor range. In general, removing respiratory regressors led to a larger decrease in ICC than removing cardiac ones. On the stringent side of the denoising spectrum, we observed that the*PNM/PNM + CSF/PNM + Moco/PNM + Moco + CSF*strategies exhibited a good to excellent stability for VV, DD, and B, while they were in the fair range for W.

To evaluate the temporal stability of noise estimates, we also computed ICC values for CSF, respiratory and cardiac time series, which all fell in the excellent range. Specifically, cardiac signals exhibited the highest ICC score with 0.95 (confidence interval at 95%, CI95% [0.96, 0.98]), followed by the respiratory signals with 0.89 (CI95% [0.75, 0.95]), and finally the CSF ICC value was 0.87 (CI95% [0.72, 0.94]).

## 4 Discussion

In recent years, a growing body of evidence has highlighted distinct spatial patterns of spontaneous activity within the human spinal cord at rest, consistently revealing correlations between its horns (Barry et al., 2014,^2018^;Eippert, Kong, Winkler, et al., 2017;Kaptan et al., 2023;Kinany et al., 2023;Kong et al., 2014). However, while these analyses have provided insights into the functional architecture of the cervical region of the spinal cord, a significant gap remains in the examination of such patterns within the lumbosacral area. In this study, we tackled this by systematically investigating horn-to-horn functional connectivity in the lumbosacral spinal cord of healthy participants. We first demonstrated that, akin to the cervical spinal cord, characteristic connectivity patterns can be identified in the lumbosacral region. In a subsequent step, we assessed the impact of different denoising strategies on these functional connectivity estimates.

### 4.1 Imaging the lumbosacral spinal cord

Despite its relevance for healthy and impaired human behavior, the human lumbosacral spinal cord has been largely unexplored in neuroimaging studies. Previous studies examining lumbosacral activity were primarily conducted during task (Jia et al., 2019;Kornelsen & Stroman, 2004,^2007^;Moffitt et al., 2005;Stroman et al., 2004), using a non-BOLD contrast mechanism known as signal enhancement from extravascular water protons (SEEP) (Stroman, Krause, Frankenstein, et al., 2001;Stroman, Krause, Malisza, et al., 2001). However, the reliability of SEEP has been a matter of debate (Bouwman et al., 2008;Jochimsen et al., 2005). Only one recent study capitalized on the BOLD signal within the lumbosacral cord during resting-state scans, providing evidence of discernible patterns of functional connectivity in the spinal cord (Combes et al., 2023).

Several distinctions between cervical and lumbosacral imaging are noteworthy. One primary concern is the marked size difference. The cross-sectional dimensions and lengths of lumbosacral segments are notably smaller (7.7 ± 2.2 mm in average) compared with the more extensively studied cervical region (13.3 ± 2.2 mm) (Frostell et al., 2016;Kinany et al., 2023). Furthermore, the anatomical positioning of both regions implies the presence of different organs in their vicinity, potentially rendering them differentially susceptible to physiological noise. Interestingly, it has been suggested that lumbosacral regions are less prone to cardiac-related motion artifacts (Figley et al., 2008).

In addition to its smaller size, the lumbosacral cord is also characterized by high intersubject variability (Tins & Balain, 2016;Van Schoor et al., 2015), marked by substantial shifts between spinal segments and vertebrae, thus making conventional normalization based on vertebral landmarks suboptimal.

### 4.2 Extension of static functional connectivity to the lumbosacral cord

In order to extend prior investigations of functional connectivity to the lumbosacral spinal cord, we resorted to established analysis techniques, commonly employed in the cervical spinal cord (Barry et al., 2014,^2018^;Eippert, Kong, Winkler, et al., 2017;Kaptan et al., 2023;Kinany et al., 2023;Kong et al., 2014). Through a ROI-based approach and using a standard denoising strategy (PNM + Moco + CSF), we demonstrated significant functional connectivity between bilateral ventral horns, bilateral dorsal horns, and between contralateral dorsoventral hemicords. A subsequent slice-wise ROI-to-voxels analysis, similar to the approach employed byBarry et al. (2016), further underscored the presence of these sensory and motor networks. This corroborates the findings ofCombes et al. (2023). However, it is noteworthy that the connectivity estimates reported in their study demonstrated a stronger amplitude than our observations, as well as the presence of significant connectivity within hemicord. These differences might be attributed to their use of trilinear interpolation, which is known to augment spatial smoothness in the dataset, could possibly contribute to the observed inflation in correlation values (Eippert, Kong, Jenkinson, et al., 2017).

The observation of bilateral connectivity patterns echoes prior investigations in the cervical spinal cord, where it has been repeatedly documented, both in animal models (L. M. Chen et al., 2015;Wu et al., 2018,^2019^) and in human studies that used various processing and acquisition procedures (Barry et al., 2014,^2018^;Eippert, Kong, Winkler, et al., 2017;Kaptan et al., 2023;Weber et al., 2018). Ventral and dorsal networks are known to be involved in motor and sensory processing, respectively (Kinany et al., 2022;Landelle et al., 2021). These bilateral networks are postulated to arise from commissural interneurons connecting neurons from the two hemicords (Maxwell & Soteropoulos, 2020). Ventral networks may serve multiple functions such as maintaining basal muscle tone—a state where motoneurons uphold posture and muscle tonicity even during quiescence (Latash & Zatsiorsky, 2015). In the lumbosacral spinal cord, which innervates lower limb muscles, these patterns may also be indicative of activity related to central pattern generators, pivotal in orchestrating locomotion (Grillner & Jessell, 2009).

Finally, we did not observe significant connectivity within (i.e., dorsoventral) hemicords, using this standard denoising strategy (PNM + Moco + CSF). Such patterns have been observed in the lumbosacral (Combes et al., 2023) and cervical spinal cord (Eippert, Kong, Winkler, et al., 2017;Kaptan et al., 2023;Weber et al., 2018). While these dorsoventral connections may potentially support polysynaptic spinal reflexes (Sandrini et al., 2005), it is important to emphasize that they seem to be strongly influenced by the specific processing approach (Eippert, Kong, Jenkinson, et al., 2017;Kaptan et al., 2023) and have exhibited poor reliability (Kaptan et al., 2023). Our subsequent investigations using distinct denoising pipelines appear to support these conclusions.

### 4.3 Impact of denoising on signal quality

Given that lumbosacral BOLD imaging has been virtually unexplored, a primary contribution of this study was the systematic evaluation of diverse denoising strategies on the time series. This comprehensive assessment included the examination of both variance explained (i.e., adjusted R^2^) and their impact on the temporal signal-to-noise ratio (tSNR).

Notably, a progressive enhancement in tSNR was observed with more rigorous denoising. This trend was mirrored in the adjusted R^2^, with the exception of the*Cardiac*pipeline, which exhibited limited explanatory power, hinting at a restricted influence of cardiac physiological noise in this spinal cord region. We could argue that the lumbar region is relatively spared from the influence of cardiac signals due to its anatomical distance from the heart, shielding it from the pulsating movement. From an anatomical point of view, the heart overlaps more with the upper regions of the spinal cord, and its upward beating within the respiratory cage may have a more pronounced impact on the cervical region compared with the lumbar region. These considerations are in line with work reporting limited cardiac-related motion in the caudal part of the spinal cord (Figley et al., 2008).

Of particular interest was the pivotal role of*PNM*regressors in improving the tSNR, underscoring their efficacy in capturing the variance of the signal. In contrast, using*CompCor*regressors to account for physiological noise yielded a more moderate effect on signal quality. These results suggest that accounting for the interaction between cardiac and respiratory signals is valuable. Besides, it hints at the fact that, despite the position of the lumbosacral region, relatively distant from the heart and lungs, mitigating potential physiological signals remains beneficial. Indeed, even in the brain, physiological fluctuations can induce notable change in fMRI time series, shown to lead to “physiological networks” (J. E. Chen et al., 2020) reminiscent of large-scale networks conventionally attributed to distantly synchronized neuronal activity.

In light of these results, our recommendation is to adopt a denoising pipeline that incorporates PNM regressors to achieve optimal enhancement of signal quality. This is in agreement with observations in the cervical spinal cord (Brooks et al., 2008;Kong et al., 2012), where PNM was found to be an effective denoising strategy, notably by eliminating false-positive activations, such as active voxels in the CSF space surrounding the cord.

### 4.4 Robustness and temporal stability of functional connectivity

Since our work primarily centered on functional connectivity, our subsequent objective was to evaluate the extent to which the strength and temporal stability of connectivity patterns were influenced by the denoising procedures applied.

While our analyses revealed a general decrease in functional connectivity with more stringent denoising, bilateral ventral (VV) and dorsal (DD) networks, as well as between-hemicords (B) connectivity, appeared to be significant regardless of the deployed denoising. This supports the genuine nature of these motor networks, emphasizing their robustness. Conversely, within hemicords (W), connectivity patterns were only significant using 8 out of 12 denoising strategies. In particular, incorporating motion parameters and physiological noise (PNM or CompCor) as regressors led to nonsignificant connectivity estimates.

To further explore the stability of these connectivity estimates, we deployed a split-half analysis to evaluate intraclass correlation coefficients (ICC). Reassuringly, DD, VV, and B connections appeared to be reliable, with ICC values primarily scoring as good (ICCs > 0.6). In comparison, cervical networks obtained using correlation analyses have been shown to exhibit a fair to good level of reproducibility, both at 7T (Barry et al., 2016) and 3T (Kaptan et al., 2023) field strengths. Likewise, similar levels of stability were reported for networks retrieved using independent component analysis (Kong et al., 2014).

Upon closer examination of the relationship between denoising strategies and the stability of functional connectivity, we did not observe a consistent decrease in stability with an increasing number of regressors, unlike recent findings in the cervical spinal cord (Kaptan et al., 2023). Instead, we noted that the removal of CompCorregressors had the most significant impact on stability. We posit that the sensitivity of the lumbosacral signals to the removal of CSF-derived principal components may pertain to the substantial volume of the subarachnoid space in these segments. Specifically, the relative average area of the spinal cord in relation to CSF in the lumbosacral cord is 29.4%, as opposed to 52.5% in the cervical region (percentages determined by calculating the average cross-sectional area of the PAM50 masks at the respective levels). This may imply additional CSF-related motion in the caudal region of the cord. Provided that CSF signals seem to exhibit structured properties, as evidenced by their ICC score in the excellent range, the CSF pipeline may impact the temporal stability by removing a large portion of reliable artifactual signals. In comparison, even though they also demonstrated high stability, physiological signals—particularly the cardiac ones—may have a comparatively lesser impact on FC stability, owing to their more limited influence on these segments of the cord.

Meanwhile, we observed that PNM-based pipelines led to robust patterns of connectivity, achieving ratings falling mostly in the good range for VV, DD, and B connections. Considering the substantial portion of variance removed by these denoising techniques (as indicated by the adjusted R^2^), this suggests that they may be a good approach to eliminate nuisance signals while preserving meaningful functional connectivity. Notably, even when combined with CSF regressors (*PNM + CSF*or the standard*PNM + Moco + CSF*), VV connectivity remained within the good range. Furthermore, our results indicate that connectivity patterns captured in split-half datasets are in agreement with those derived from the full dataset, supporting the use of runs shorter than 10 min to estimate lumbosacral resting-state functional connectivity.

### 4.5 Limitations of the study

The current study has several limitations that warrant acknowledgment. First, we could not define ROIs directly in the native space, due to the limited spatial resolution of the functional images. Instead, ROIs were defined using the PAM50 atlas warped into the functional space of each participant. Even though the normalization procedure appeared to be accurate, this method may have resulted in underestimated connectivity estimates. Future work could focus on optimizing acquisition parameters to achieve a higher contrast between gray and white matter in the functional scans. Alternatively, acquiring additional high-resolution T2*-weighted images could provide better delineation of the different horns by facilitating gray matter segmentation. Second, the temporal stability estimates were derived from split-half time series, potentially leading to inflated values compared with those obtained from separate test–retest runs, despite the length of the runs (15 min in total). Employing distinct test–retest sessions could offer a more accurate reflection of the reliability of functional connectivity patterns. Third, we did not investigate intersegmental functional connectivity estimates. Given the small size of the lumbosacral segments, this effort may require the acquisition of additional anatomical images with sufficient resolution to precisely identify the nerve roots and, thus, the spinal segments (Rowald et al., 2022) Finally, the present results do not offer direct insights into the mechanisms driving the observed correlations. To address this limitation, future research endeavors should consider integrating behavioral or clinical data alongside functional imaging.

### 4.6 Conclusion and outlook

In summary, our findings underscore the existence of intrinsic functional connectivity in the lumbosacral region, in the form of bilateral ventral connectivity. Importantly, the robustness of these connectivity patterns was confirmed by their persistence across various denoising strategies. In addition, our results hint at the effectiveness of physiological noise modeling (PNM) as a valuable approach for denoising lumbosacral spinal cord fMRI images, while preserving the strength and stability of functional connectivity estimates. Finally, given the nascent stage of lumbosacral fMRI research, future investigations are needed to probe these findings across diverse acquisition and processing schemes. While the current study proposes a first step in this direction, further research is necessary to ascertain the robustness and broader applicability of the observed functional connectivity patterns in the lumbosacral spinal cord.

## Supplementary Material

Supplementary Material

## Data Availability

Code is publicly available on GitHub (https://github.com/iricchi/Lumbar.git). The data can be accessed on Mendeley Data with the identifier doi: [to be done upon acceptance].
