# Supplementary material for: Lumbosacral spinal cord functional connectivity at rest: From feasibility to reliability
Source: Imaging Neurosci (Camb). 2024 Sep 5;2:imag-2-00286. doi: 10.1162/imag_a_00286 (PMC12290568; doi:10.1162/imag_a_00286)
Supplement: Supplementary Material [file imag_a_00286-supp.pdf]

Supplementary material

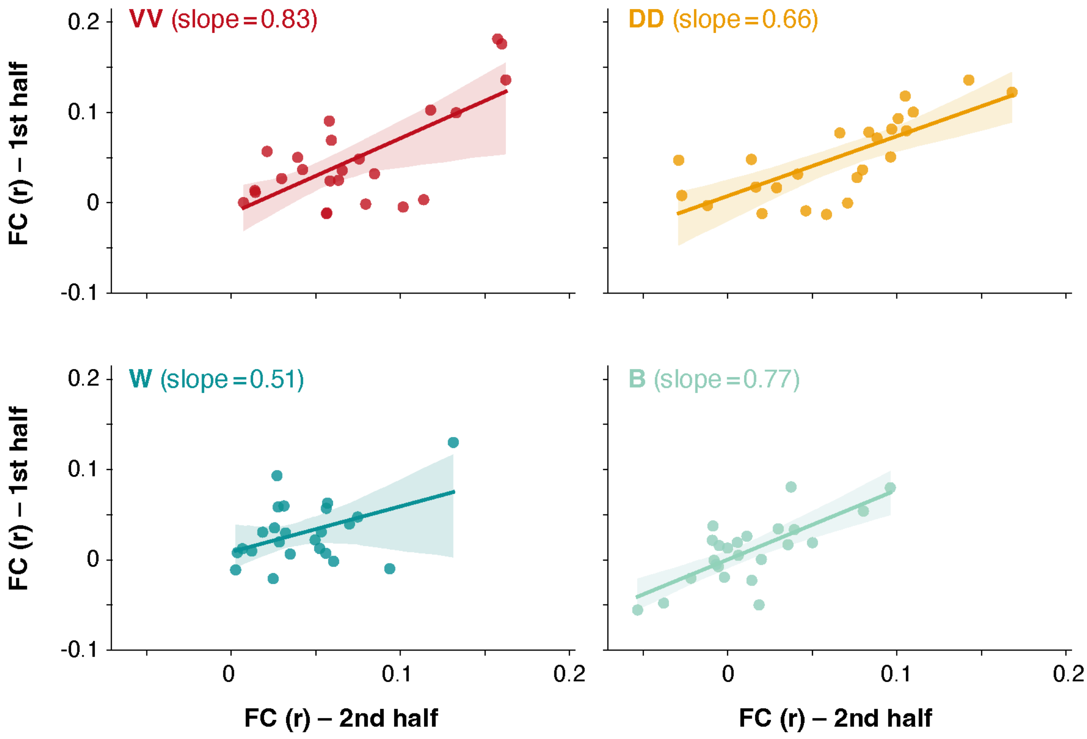

Figure S1. FC temporal stability. Scatter plots illustrate the correlation values in each half dataset plotted against each other, with the x-axis representing the first half and the y-axis representing the second half). Correlations values are obtained using the time courses denoised with the *PNM+Moco+CSF* pipeline. Ventral-ventral (VV) connectivity is depicted in red, dorsal-dorsal (DD) in yellow, within horns in dark green, and between horns in light green. The slopes for VV, DD, within horns (W), and between horns (B) are 0.83, 0.66, 0.51, and 0.77, respectively.

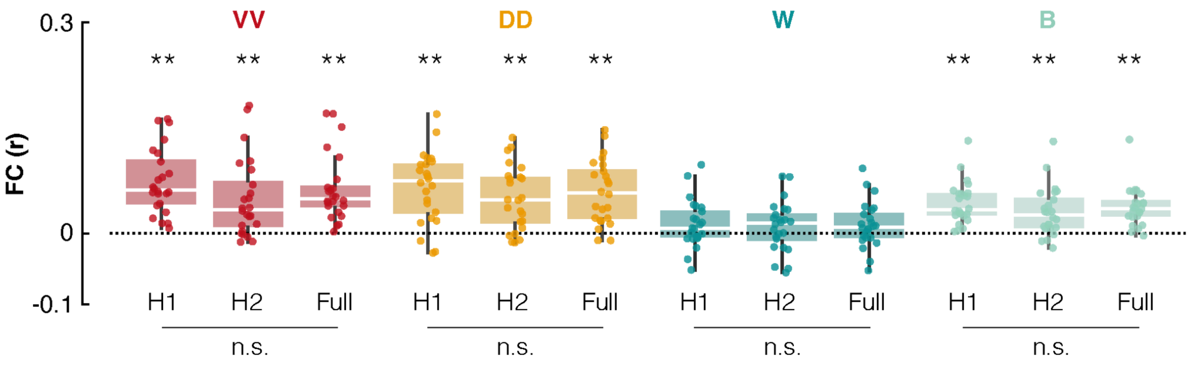

Figure S2. Split-half functional connectivity. We present functional connectivity estimates (y-axis) for split-halves (H1 and H2) of the data, as well as for the full dataset (x-axis). Correlations values are obtained using the time courses denoised with the PNM+Moco+CSF pipeline. Ventral-ventral (VV)

connectivity is depicted in red, dorsal-dorsal (DD) in yellow, within horns in dark green, and between horns in light green. The boxes represent the interquartile range (IQR), spanning from the 25th to the 75th percentile, with the horizontal white line within each box indicating the median value across participants. Each dot represents the mean FC (across slices) for a specific participant. ** indicates $p < 0.01$. n.s. stands for non-significant (i.e., no statistical difference across halves and full dataset).
